# Research progress on intraocular lens power calculation in cataract patients with prior radial keratotomy

**DOI:** 10.3389/fmed.2026.1878452

**Published:** 2026-07-20

**Authors:** Qian Zhao, Ziqi Liang, Ling Peng, Wenqun Xi, Xinya Hu, Yu Zhang, Kun Zeng, Zhe Zhang

**Affiliations:** Department of Ophthalmology, Shenzhen Eye Hospital, Shenzhen Eye Medical Center, Southern Medical University, Shenzhen, China

**Keywords:** cataract, intraocular lens calculation, light adjustable lens, radial keratotomy, refractive outcome

## Abstract

Radial keratotomy (RK) was a refractive procedure widely used to correct myopia from the 1980s to the 1990s. Although this technique has been gradually replaced by modern laser corneal refractive surgery, a large number of post-RK patients are now entering their senior years and requiring cataract surgery. The altered corneal morphology after RK poses unique challenges for intraocular lens (IOL) power calculation, including changes in the relationship between anterior and posterior corneal curvatures, difficulty in predicting effective lens position, irregular corneal astigmatism, and diurnal fluctuation of refraction. In recent years, with the development of new-generation IOL calculation formulas, artificial intelligence algorithms, and adjustable IOL technologies, the refractive outcomes of cataract surgery in post-RK patients have significantly improved. This narrative review summarizes the changes in corneal biomechanical and optical properties after RK, evaluates the reported accuracy of existing IOL formulas for post-RK eyes, discusses intraoperative considerations and postoperative complication management, and prospects the application of novel adjustable IOLs and small-aperture IOLs in this patient population.

## 1 Introduction

Radial keratotomy was popularized by Russian ophthalmologist Fyodorov in the 1970s. By making symmetric radial incisions in the mid-peripheral cornea, the peripheral cornea bulges and the central cornea flattens, thereby reducing corneal power and correcting myopia (1). It is estimated that millions of patients worldwide have undergone RK. Although this procedure achieved satisfactory visual outcomes in its era, its long-term stability, predictability, and safety have been controversial. The 10-year follow-up results of the Prospective Evaluation of Radial Keratotomy (PERK) study showed that 43% of patients experienced overcorrection of more than 1.0 D, and this hyperopic shift progressed over time at a rate of approximately 0.06 D per year (2).

As post-RK patients age naturally, cataract has become the leading cause of visual impairment in this population. However, the altered corneal morphology after RK makes it difficult for conventional IOL formulas to accurately predict postoperative refraction. Major issues include: inaccurate corneal power measurement leading to errors in corneal power estimation; altered ratio of anterior to posterior corneal curvature rendering the traditional keratometric index invalid; bias in effective lens position prediction; and irregular astigmatism and diurnal refractive fluctuations (3). Studies have shown that using conventional IOL formulas for post-RK eyes, only about 50% of eyes achieve a target refraction within ±0.5 D (4).

In recent years, with the emergence of fourth- and fifth-generation IOL formulas and the introduction of artificial intelligence, the accuracy of IOL calculation in post-RK eyes has improved. Meanwhile, novel IOLs such as the light adjustable lens (LAL) and small-aperture IOL offer the possibility of postoperative fine-tuning for these patients. This article provides a narrative review of these advances. Recent comprehensive reviews have addressed IOL calculation in eyes after various types of corneal refractive surgery, including LASIK, PRK, and RK (5). However, the unique challenges posed specifically by radial keratotomy—including irregular astigmatism, altered anterior–posterior corneal curvature relationships, and diurnal refractive fluctuations—warrant a focused review dedicated solely to this population.

Compared with previous reviews, our distinctive contributions include: (1) systematic summary of the newest formulas (VRF, PEARL-DGS, Jin-AI) in post-RK eyes; (2) inclusion of evidence on small-aperture IOLs and LAL specifically for RK patients; (3) discussion of angle kappa-guided keratometry mode selection and total keratometry applications; and (4) a practical clinical algorithm.

## 2 Literature search methods

Because this is a narrative review, we did not perform a formal systematic meta-analysis. However, the PRISMA (Preferred Reporting Items for Systematic Reviews and Meta-Analyses) framework was used as a guiding structure for the literature search and study selection process to ensure methodological transparency. To ensure comprehensive coverage, we conducted a literature search in PubMed, Web of Science, and Scopus up to May 2026 using relevant keywords related to radial keratotomy, cataract, and intraocular lens power calculation. Additional studies were identified by hand-searching the reference lists of included articles and relevant reviews.

### 2.1 Inclusion and exclusion criteria

Studies were included if they met all of the following criteria: (1) original research articles published in peer-reviewed journals; (2) studies related to cataract management in eyes with prior radial keratotomy (RK), including but not limited to IOL power calculation formula accuracy, corneal pathophysiological changes, intraoperative considerations, and novel IOL technologies; (3) reporting original data or providing novel technical insights.

**Figure 1:**
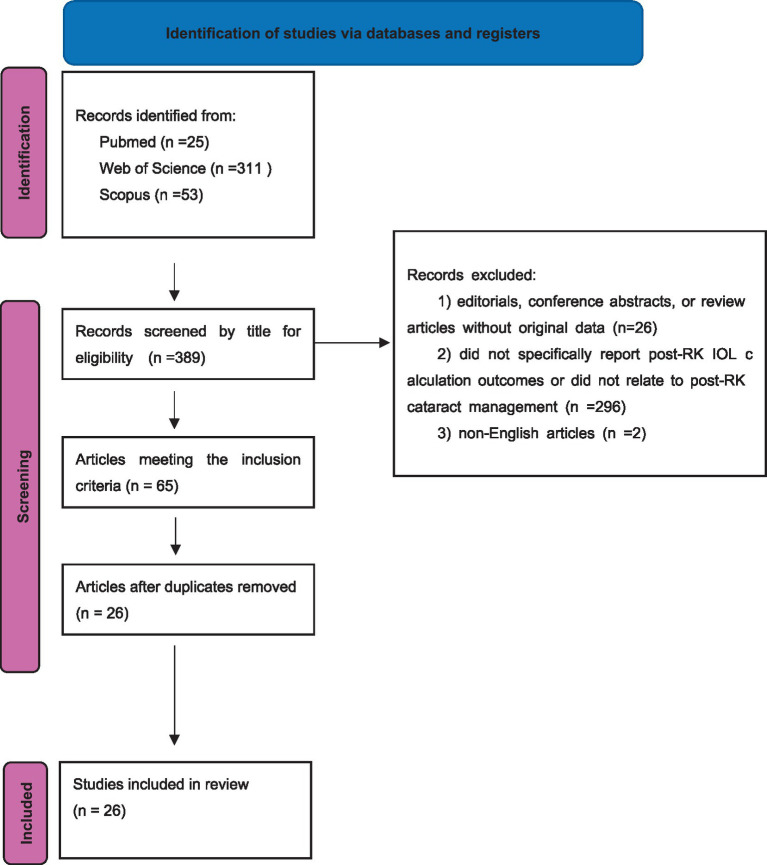
PRISMA flow diagram of the literature search and selection process.

Studies were excluded if they: (1) were editorials, conference abstracts, or review articles without original data; (2) did not specifically report post-RK IOL calculation outcomes or relate to post-RK cataract management; (3) non-English articles. This process yielded 26 relevant studies that form the basis of this review (Figure 1).

## 3 Pathophysiological characteristics of the post-RK cornea

### 3.1 Corneal morphological changes

RK surgery involves making 4, 8, 12, or up to 50 radial incisions in the mid-peripheral cornea, cutting collagen fibers in the corneal stroma and disrupting the original biomechanical balance (6). The postoperative cornea exhibits a characteristic “central flattening, peripheral steepening” oblate shape, with significantly reduced central optic zone curvature. Tsai et al. (7) reported a rare case of central corneal steepening and myopic shift in a post-RK patient. Corneal topography showed mean central corneal curvatures of 44.8 D (right eye) and 42.7 D (left eye). Anterior segment OCT revealed subepithelial elevated hyperreflective material in the central cornea; after superficial keratectomy, corneal curvatures decreased to 40.5 D and 36.2 D, respectively, confirming that surface pathology can influence curvature. This case suggests that late myopic shift after RK may originate from corneal surface lesions rather than simple corneal ectasia.

### 3.2 Altered relationship between anterior and posterior corneal curvatures

In unoperated normal eyes, the anterior and posterior corneal curvature radii have a relatively fixed ratio (approximately 1.2:1). Conventional keratometers use a fixed keratometric index (1.3375) to convert anterior curvature to total corneal power (8). RK surgery alters not only the anterior curvature but also the posterior surface morphology. Camellin et al. (9) used Scheimpflug camera measurements and found that the ratio of posterior to anterior corneal curvature radius changes significantly after RK, rendering the traditional keratometric index invalid—a major source of error in IOL formulas.

### 3.3 Diurnal refractive fluctuation

One of the most concerning complications after RK is diurnal fluctuation of refraction. Bullimore et al. (10) studied 10 firefighter applicants 4–14 months after RK and found a mean myopic shift of −0.41 D from morning to afternoon, highly correlated with significant corneal steepening (+0.41 D, *r* = −0.86). Long-term follow-up of the PERK study further confirmed that 10 years after surgery, RK eyes still exhibited significant diurnal refractive fluctuation, with a mean myopic shift of 0.36 D, correlated with increased mean corneal power (0.52 D) and changes in intraocular pressure (11). The mechanism of this diurnal fluctuation may relate to the dynamic process of corneal edema during sleep (eyelids closed, hypoxic environment, increased stromal water, corneal thickening) and corneal dehydration after waking. The healing tissue at RK incisions has different compliance than normal cornea, making the cornea more susceptible to curvature changes during this process (10).

### 3.4 Increased higher-order aberrations

The irregular corneal shape after RK leads to a significant increase in higher-order aberrations. Applegate et al. (12) showed that post-RK eyes mainly exhibit increased spherical aberration and coma, with the magnitude of aberration related to optic zone diameter and number of incisions. Van den Berg et al. (13) studied 32 post-RK cataract patients and found that the root mean square of corneal higher-order aberrations under natural pupils (mean 3.0 mm) was (0.185 ± 0.029) μm, significantly higher than normal controls. Increased higher-order aberrations not only affect uncorrected visual acuity but also increase inaccuracies in IOL power measurement.

## 4 Research progress on IOL calculation formulas for post-RK eyes

### 4.1 Limitations of traditional formulas

Early studies used third-generation formulas such as SRK/T, Hoffer Q, and Holladay I to calculate IOL power for post-RK eyes, and hyperopic shift was commonly observed. Chen et al. (14) found that using standard keratometry values and conventional formulas, post-RK eyes had a mean hyperopic prediction error of approximately +1.0 D. This is because the true corneal power is overestimated after RK, leading to underestimation of IOL power. This finding is corroborated by Helaly et al. (15), who reported that third-generation formulas (SRK/T and Holladay I) yielded the highest MAE (1.62 D and 1.42 D, respectively) among 14 formulas evaluated in 50 post-RK eyes.

### 4.2 Modified history-based formulas

#### 4.2.1 Barrett True-K formula

The Barrett True-K formula is currently recognized as one of the preferred formulas for IOL calculation in post-RK eyes. It uses the theoretical framework of the Barrett Universal II formula and incorporates pre- and postoperative refractive data to back-calculate the true corneal power (16). Turnbull et al. (3) studied 52 post-RK eyes and found that the median absolute error (MedAE) of Barrett True-K with full history was 0.33 D, with 76.6% of eyes within ±0.5 D of target refraction; for history-independent Barrett True-K, MedAE was 0.47 D, with 69.2% within ±0.5 D. Li et al. (17) compared the accuracy of Barrett True-K, Haigis, and Holladay I D-K formulas in a Chinese population and found that Barrett True-K had the lowest MedAE (0.620 D), with 46.81% of eyes having absolute error within 0.5 D. That study also found that axial length was significantly correlated with prediction error, with longer eyes (>26 mm) showing larger errors. More recently, Li et al. (18) confirmed the superiority of Barrett True-K in a 72-eye Chinese cohort, reporting the lowest MAE (0.84 D) and MedAE (0.65 D) among five modern formulas.

#### 4.2.2 Shammas and Haigis-L formulas

The Shammas formula and Haigis-L formula are history-independent methods designed for post-refractive surgery eyes. They compensate for corneal power overestimation after RK by adjusting the keratometric index or using regression formulas (19). In a multicenter study by Moshirfar et al. (20) including 37 post-RK eyes, Haigis-L had a MedAE of 0.629 D and Shammas formula a MedAE of 0.650 D, both slightly inferior to the average result of the ASCRS online calculator (0.535 D).

### 4.3 New-generation artificial intelligence formulas

#### 4.3.1 Kane formula

The Kane formula is a new IOL calculation formula based on theoretical optics combined with multivariate regression and artificial intelligence components. Cheng et al. (21) showed that the Kane formula has high predictive accuracy in the general cataract population. In post-RK eyes, Ferrara et al. (4) studied 27 post-RK eyes and found that the Kane formula had a mean absolute error (MAE) of 0.19 ± 0.14 D, with 74.1% of eyes within 0.25 D. In that single study, Kane performed better than SRK-T and Barrett True-K. Moshirfar et al. (20) in a multicenter study also confirmed that the Kane formula achieved 48.65% of eyes within ±0.5 D and 75.68% within ±1.0 D, with a MedAE of 0.555 D.

#### 4.3.2 PEARL-DGS formula

PEARL-DGS (Prediction Enhanced by Artificial Intelligence and output Linearization-Debellemaniere, Gatinel, and Saad) is an open-sourece IOL calculation formula developed using machine learning algorithms. It employs a thick-lens model and optimizes multiple biometric inputs (22). In the study by Ferrara et al. (4) on 27 post-RK eyes, PEARL-DGS achieved an MAE of 0.17 ± 0.14 D, with 77.8% of eyes within 0.25 D – the best among the five formulas tested in that particular cohort. In a larger cohort of 50 post-RK eyes, Helaly et al. (15) further evaluated PEARL-DGS and found that it performed particularly well in eyes with flat keratometry (<38 D), achieving an MAE of 0.72 D and 44% of eyes within ±0.50 D. Of note, this was the first study to report the performance of PEARL-DGS specifically in post-RK eyes with flat corneas.

#### 4.3.3 EVO 2.0 formula

EVO 2.0 (Emmetropia Verifying Optical) formula is based on the emmetropization theory of the optical system, calculating IOL power by determining an “emmetropia factor” (23). In the study by Moshirfar et al. (20), EVO 2.0 had a MedAE of 0.545 D, with 48.65% of eyes within ±0.5 D and 64.86% within ±0.75 D. This formula performed particularly stably in eyes with medium axial lengths.

#### 4.3.4 VRF formula

The VRF formula was developed by Voytsekhivskyy in 2018. Its innovation lies in using four predictive variables (axial length, corneal curvature, preoperative anterior chamber depth, and corneal diameter) to predict effective lens position (24). The original study included 823 normal cataract patients and showed that the VRF formula had a MedAE of 0.305 D across all axial lengths, outperforming Haigis (0.329 D) and Holladay I (0.326 D). Guo et al. (25) first applied the VRF formula to post-RK cataract patients in a retrospective study of 50 eyes (27 patients). In that cohort, the VRF formula had a MedAE of 0.580 D, with 48% of eyes within ±0.5 D, 66% within ±0.75 D, and 78% within ±1.0 D. Mean prediction error analysis showed that only VRF formula had a mean prediction error (0.150 D) not significantly different from zero (*p* = 0.483), while Kane (0.748 D) and Barrett True-K (−0.565 D) exhibited systematic bias. These findings are promising but need replication.

#### 4.3.5 Jin-AI formula

The Jin-AI formula is a novel artificial intelligence-based IOL power calculation method trained specifically on Chinese eyes. It employs a hybrid approach that combines a multilayer neural network (with three hidden layers) to predict patient-specific effective lens position, followed by standard thin-lens vergence calculation (Gaussian optics) to derive the final IOL power recommendation (26). Xiang et al. (27) recently evaluated the accuracy of eight IOL formulas, including Jin-AI, in 34 Chinese post-RK eyes. The Jin-AI formula achieved the lowest MedAE (0.49 D) and the highest percentage of eyes within ±0.50 D (47.1%), comparable to Barrett True-K (MedAE 0.535 D, 44.1% within ±0.50 D) and EVO (MedAE 0.538 D, 44.1% within ±0.50 D). The authors concluded that Jin-AI represents a promising option for post-RK IOL calculation in Chinese populations, although independent validation in non-Chinese cohorts is still needed. This finding underscores the potential value of ethnic-specific AI models in addressing the unique biometric characteristics of Chinese eyes, such as shallower anterior chamber depth and lower keratometric index compared to Caucasian eyes.

### 4.4 Selection of keratometry measurement mode

Accurate keratometry is critical for IOL calculation. Inaccurate keratometry measurement accounts for up to 22% of IOL power calculation errors in challenging eyes (28). For post-RK eyes, this proportion may be even higher due to altered anterior–posterior curvature relationships. Therefore, optimizing keratometry mode (e.g., considering angle kappa, using total keratometry when available) is essential. As shown by Zheng et al. (29), total keratometry can modestly improve prediction accuracy for some formulas, but the benefit is not universal. Yuan et al. (30) studied post-refractive surgery eyes (LASIK/PRK) and found that angle kappa significantly influenced predictive accuracy. When kappa ≥ 0.4 mm, corneal curvature values centered on the corneal vertex (IOLMaster 700 and Pentacam CV mode) produced hyperopic bias, while pupil-centered corneal curvature values (Pentacam PC mode) had no systematic bias. Although that study did not include RK eyes, the underlying principle (angle kappa-induced decentration) may also apply to RK because RK similarly alters central-peripheral curvature relationships. Therefore, for post-RK patients with large angle kappa, pupil-centered keratometry could be considered, but RK-specific validation is needed.

### 4.5 Total keratometry in post-RK eyes

Recent advances in swept-source optical biometry allow measurement of total keratometry (TK), which incorporates both anterior and posterior corneal curvatures. Zheng et al. (29) evaluated the performance of IOL power calculation formulas using standard keratometry (SK) versus TK in 65 post-RK eyes. Among formulas using SK, the LISA-MRS formula exhibited the lowest MAE (0.71 D) and MedAE (0.52 D), with 49.23% of eyes within ±0.5 D. For formulas using TK, the LISA-MRS-TK formula showed the lowest MAE (0.78 D) and MedAE (0.48 D), with 52.63% within ±0.5 D. Importantly, using TK instead of SK significantly reduced the prediction error of the Holladay 2 and LISA-MRS formulas. The authors concluded that TK can improve prediction accuracy for certain formulas in post-RK eyes, although the absolute gains were modest. This study highlights the potential value of incorporating posterior corneal curvature measurements, which is particularly relevant given the altered anterior–posterior curvature ratio after RK.

Table 1 summarizes the principles, advantages, limitations, and clinical indications of the major IOL formulas for post-RK eyes discussed above.

**Table 1:** Comparison of major IOL formulas for post-RK eyes.

Formula	Principle/algorithm	Advantages in post-RK	Limitations in post-RK	Clinical indication
Barrett True-K	Theoretical optics + history-based K adjustment	Full-history: MedAE 0.33 D, 76.6% within ±0.5 D	Requires full refractive history; no-history version less accurate (MedAE 0.47 D); larger errors in eyes with AL > 26 mm	First choice when full history available
Kane	Hybrid (theoretical optics + multivariate regression + AI components)	Low MAE (0.19 ± 0.14 D) in one small study; high accuracy in general cataract population	Small sample validation; inconsistent performance across cohorts	Alternative when history unavailable; use in combination with other formulas
PEARL-DGS	Open-source machine learning, thick-lens model	Lowest MAE (0.17 ± 0.14 D) and 77.8% within ±0.25 D in single 27-eye series	Only one small study; lacks external validation; performs well in flat corneas (<38 D) but not specifically optimized for all RK eyes	Investigational; requires replication
EVO 2.0	Emmetropization theory (“emmetropia factor”)	Stable performance in medium axial lengths; history-independent	Moderate accuracy (MedAE 0.545 D, 48.65% within ±0.5 D); limited post-RK data	One of multiple formulas for result averaging
VRF	Four-variable ELP prediction (AL, K, preoperative ACD, corneal diameter)	No systematic bias in 50-eye study (mean PE 0.150 D, *p* = 0.483)	Moderate accuracy (MedAE 0.580 D, 48% within ±0.5 D); requires dedicated software	Consider when history unavailable and other options limited
LISA-MRS	Ensemble learning, thick-lens, designed for post-refractive eyes	Lowest MAE (0.71 D) and MedAE (0.52 D) in Chinese cohort; TK further improves accuracy	Validated only in Chinese population; modest absolute gain with TK	For Chinese patients, especially when TK available
Jin-AI	Hybrid AI (neural network + Gaussian optics), trained on Chinese eyes	Lowest MedAE (0.49 D) and 47.1% within ±0.5 D in Chinese cohort	Validated only in Chinese eyes; external validation needed	For Chinese patients when available

Direct cross-study comparisons are invalid due to heterogeneity in sample size, biometers, and outcome metrics. Values shown are from original studies and should be interpreted within their specific cohort contexts.

## 5 Application prospects of small-aperture IOLs

Van Den Berg et al. (13) investigated the potential of a small-aperture IOL (IC-8^®^ Aphera™) in post-RK patients. This IOL features an embedded pinhole aperture of 1.36 mm (corresponding to 1.6 mm at the corneal plane), increasing depth of focus and reducing the impact of higher-order aberrations via the pinhole effect. The study included 32 post-RK cataract patients. Predictive analysis showed that with a 1.6 mm aperture, the root mean square of corneal higher-order aberrations decreased from (0.185 ± 0.029) μm under natural pupils to (0.063 ± 0.015) μm, a reduction of 66%. A representative case showed that postoperative uncorrected distance visual acuity improved from 20/100 to 20/30 + 2, uncorrected near visual acuity reached 20/12, and higher-order aberrations decreased by 75%.

The advantage of small-aperture IOLs is that they provide good functional vision without relying on accurate keratometry, making them particularly suitable for post-RK patients with highly irregular astigmatism. However, limitations include restricted visualization of the peripheral retina, risk of damage during Nd: YAG laser capsulotomy, and possible decreased contrast sensitivity under dim lighting (31).

## 6 Progress in light adjustable Lens application

### 6.1 Technical principles of LAL

The light adjustable lens (LAL) is a postoperatively adjustable IOL made of photosensitive silicone material (32). Its basic principle is that the IOL contains photosensitive macromers that polymerize upon exposure to a specific wavelength of ultraviolet light, changing the IOL curvature and thus adjusting refraction. The second-generation LAL adds a UV-blocking layer to reduce accidental environmental UV exposure to unlocked lenses (33). LAL can adjust sphere by ±2.0 D and cylinder up to −2.0 D, typically requiring 2–3 adjustments and 2 lock-in treatments, with the entire process starting 6–8 weeks postoperatively and lasting about 1 month. Patients must wear dedicated UV-protective eyewear during this period (32).

### 6.2 Application of LAL in post-RK patients

Webster et al. (34) reported the largest case series to date (94 eyes) of LAL implantation in post-RK eyes. Results showed that 82% of eyes achieved uncorrected distance visual acuity of 20/25 or better, and 74% achieved 20/20 or better; 98% of eyes were within ±1.0 D of target refraction, and 88% within ±0.5 D. An important finding was that delaying the first light adjustment until 6–8 weeks postoperatively to allow sufficient corneal stabilization after RK may be key to achieving good refractive outcomes.

Jiang et al. (35) reported bilateral LAL implantation in a patient with 50-cut RK (the highest number of RK incisions reported in the literature). Preoperatively, the patient had significant irregular astigmatism (right eye −1.50 + 2.25 × 145, left eye +0.25 + 1.25 × 040). After two rounds of adjustment and lock-in, final refractions were right eye −0.25 + 0.25 × 110 (UDVA 20/20–2) and left eye −0.25 + 0.50 × 135 (UDVA 20/25–1). This case demonstrates that even for patients with very high incision counts, LAL can effectively correct residual refractive error.

Moshirfar et al. (36) compared LAL outcomes in patients with and without prior corneal refractive surgery and found no significant difference in postoperative UDVA between the two groups, but the RK group required more adjustments (mean 2.2 vs. 1.3), possibly related to refractive instability of the post-RK cornea.

### 6.3 Indications and precautions for LAL

LAL is suitable for post-RK patients who have high expectations for uncorrected vision after cataract surgery, provided that the following conditions are met: the pupil can be adequately dilated (at least 6 mm, ideally 7 mm); the patient can tolerate 3–5 light treatments; and the patient is willing to comply with UV protection requirements. Contraindications include RK incisions with obvious gaps or subepithelial fibrosis, and the presence of severe irregular astigmatism, as LAL cannot correct pre-existing corneal higher-order aberrations (34).

## 7 Discussion

### 7.1 Limitations of existing studies

A comprehensive analysis of the literature reveals several common limitations in studies on IOL calculation in post-RK eyes. First, sample sizes are generally small. Most studies have sample sizes between 30 and 50 eyes; the largest studies (Webster et al., 94 eyes; Voytsekhivskyy, 78 eyes) are still much smaller than typical studies in normal cataract patients. This is related to the decline of RK surgery and the naturally decreasing number of post-RK patients (34, 37). Second, documentation of RK surgical parameters is often incomplete. Since most RK surgeries were performed three to four decades ago, preoperative refractive data and surgical parameters (number of incisions, optic zone diameter, incision depth, etc.) are often missing, limiting the application of history-based formulas (3). Third, current formulas do not differentiate between incision number or axial length, both of which may affect postoperative refractive error. Fourth, racial differences and formula generalizability remain questionable. Most studies come from European or American populations, with relatively few studies in Chinese populations. Although the studies by Li et al. (17) and Guo et al. (25) have filled the data gap for Chinese populations, differences in IOL constant optimization and postoperative follow-up time make direct comparisons difficult. Fifth, long-term follow-up data are lacking. The post-RK cornea undergoes long-term morphological evolution, but current IOL formula studies have at most 6–12 months of follow-up; long-term stability (e.g., 5–10 years postoperatively) is unknown.

### 7.2 Comparison with general cataract population and other special populations

For normal cataract patients, modern IOL formulas achieve ±0.5 D in over 80% of eyes. A recent study reported that only 73.7% of unselected cataract patients achieve postoperative prediction error within ±0.5 D (38). In post-RK eyes, even with the best available formulas (e.g., full-history Barrett True-K or LAL), the proportion within ±0.5 D can reach 76–88% in selected series. However, when historical data are unavailable or traditional formulas are used, the success rate often falls below 50%. Thus, post-RK eyes remain a high-risk group for refractive surprise.

It is instructive to compare post-RK eyes with another challenging population: pediatric cataract patients. A systematic review by Stopyra and Grzybowski (39) found that even the best formulas (e.g., Barrett Universal II) achieve only modest accuracy in children, with MAEs around 0.8–1.0 D and substantial variability. Both populations share the problem that standard formulas developed on adult virgin eyes do not directly apply, and dedicated formula modifications or adjustments are necessary.

### 7.3 Artificial intelligence: principles and limitations

The term “AI-based formulas” often refers to the use of machine learning (e.g., multivariate adaptive regression splines, random forests, or neural networks) to model nonlinear relationships between biometric inputs and IOL power. For example, PEARL-DGS uses a machine learning algorithm to optimize coefficients for predicting effective lens position, and the Kane formula incorporates AI components to refine regression outputs. However, current AI models for post-RK eyes face significant limitations: small training samples (often <100 RK eyes), lack of external validation, and the “black box” nature that may not generalize to unusual corneas. Li et al. (18) found that EVO 2.0, Kane, and PEARL-DGS all exhibited significant hyperopic bias in post-RK eyes, suggesting that current AI-based formulas may not fully compensate for RK-induced corneal alterations. Moreover, most AI formulas were developed on normal cataract populations and then applied to RK eyes without retraining. Future AI models dedicated to post-RK eyes, incorporating anterior and posterior corneal elevation maps, are needed. The application of AI in corneal ectasia diagnosis has shown promise; for example, deep learning models trained on Scheimpflug tomography can detect keratoconus with high accuracy (40), suggesting that similar approaches could be adapted for RK-specific IOL calculation.

With advances in artificial intelligence, IOL calculation formulas based on machine learning and deep learning are expected to further improve predictive accuracy in post-RK eyes. Researchers have begun to explore neural network models that input more dimensions of biometric parameters (e.g., anterior and posterior corneal elevation maps, anterior and posterior corneal curvature maps, corneal thickness distribution maps) to obtain individualized corneal power corrections (22).

### 7.4 Future directions: AI and multimodal imaging

Future AI models dedicated to post-RK eyes should be trained on large, multicenter datasets that include anterior and posterior corneal elevation maps, corneal thickness profiles, and higher-order aberrations. The success of AI in detecting and managing keratoconus – another corneal ectatic condition – offers a roadmap. Goodman and Zhu (40) systematically reviewed 93 studies on AI for keratoconus, finding that deep learning models trained on Scheimpflug tomography achieve high diagnostic accuracy and can aid in severity grading and progression prediction. Similar approaches could be adapted for RK: for instance, a neural network could be trained to predict the “true” corneal power directly from tomographic maps, bypassing the flawed keratometric index. Until such tools are available, clinicians should use a combination of formulas (e.g., averaging outputs from Barrett True-K, Kane, VRF, and the ASCRS online calculator) and consider postoperative adjustment technologies (LAL or small-aperture IOLs) for patients at high risk of residual error.

## 8 Clinical practice recommendations

Based on the above, the following recommendations are proposed for IOL calculation and surgical management of cataract patients with prior RK, drawing on the findings of the reviewed studies:

### 8.1 First, preoperative assessment

Document RK history (if possible), including year of surgery, number of incisions, optic zone diameter, and preoperative refraction. Perform Scheimpflug tomography to evaluate anterior and posterior corneal morphology and higher-order aberrations. Measure angle kappa; for kappa ≥ 0.4 mm, consider using pupil-centered keratometry. Assess diurnal refractive fluctuations by repeating measurements at different times of the day.

### 8.2 Second, IOL formula selection

Prioritize Barrett True-K (full history version) or VRF-GL formula. If history is unavailable, choose Barrett True-K (no history version), EVO 2.0, Kane, or VRF formulas. The average result of the ASCRS online calculator can serve as a reference. For Chinese patients, Jin-AI, Barrett True-K, and LISA-MRS have shown favorable accuracy. Averaging results from multiple formulas may be better than any single formula (Table 1).

### 8.3 Third, target refraction setting

Given the progressive hyperopic shift after RK, it is recommended to set target refraction between −0.75 D and −0.5 D to provide a myopic buffer (41, 42). For younger patients or those with high demand for distance vision, a lower residual refraction may be considered.

### 8.4 Fourth, surgical techniques

For cases with a high number of incisions, prefer a scleral tunnel incision. If a clear corneal incision is necessary, make it at the widest point between two RK incisions, keep it as small as possible (2.0–2.4 mm), and consider a pre-placed suture. Meduri et al. (43) demonstrated that a precautionary 10–0 nylon stabilizing suture across the adjacent RK incision reduced the intraoperative dehiscence rate to 0% in a prospective study of 24 eyes with 16 RK incisions. A subsequent study by the same group further showed that a double-suture technique provided better preservation of corneal biomechanical properties and lower residual astigmatism compared to a single suture (44). Perform a Seidel test at the end of surgery to confirm watertight closure of all incisions.

### 8.5 Fifth, special IOL selection

For patients with high expectations for uncorrected vision and ability to tolerate multiple light treatments, LAL implantation may be considered, provided that adequate pupil dilation and postoperative UV protection compliance can be ensured. For patients with significant irregular astigmatism, a small-aperture IOL (IC-8) may provide more stable visual outcomes.

### 8.6 Sixth, postoperative management

Avoid eye rubbing and strenuous activity in the early postoperative period. For LAL recipients, delay the first light adjustment until 6–8 weeks postoperatively. YAG laser capsulotomy should be performed after refraction has stabilized (usually ≥3 months postoperatively).

## 9 Conclusion

IOL calculation for cataract patients with prior radial keratotomy remains a major challenge in refractive cataract surgery. In the past decade, with the emergence of new-generation formulas such as Barrett True-K, Kane, EVO 2.0, PEARL-DGS, and VRF, and the application of novel IOLs including LAL and small-aperture IOLs, postoperative refractive outcomes in these patients have markedly improved. However, the proportion of eyes achieving ±0.5 D varies widely depending on formula selection and availability of historical data: with full-history Barrett True-K or LAL, success rates of 76–88% have been reported; but when history is unavailable or older formulas are used, the rate often falls below 50%. This underscores that post-RK eyes remain a higher-risk group compared to the general cataract population.

As the population ages, the number of cataract patients with prior RK will continue to grow over the next decade. Future research should focus on multicenter prospective studies with standardized reporting, AI-based individualized models integrating multimodal imaging (including total keratometry and tomographic maps), and long-term follow-up to assess refractive stability. Ophthalmologists must appreciate the unique characteristics of this group and apply current tools and techniques to provide individualized precision treatment.
